# New York Medical Society and Homœophy

**Published:** 1884-11-20

**Authors:** 


### NEW YORK MEDICAL SOCIETY AND HOMŒOPATHY.

"Ye shall know them by their fruits ;" and no one should bring a charge of inconsistency against the Medical Society of the County of New York for taking Homœopaths into membership, or for laying upon the table a resolution to the effect that a diploma from a homœopathic medical college did not entitle the holders to membership in the society.

After recognizing this class of practitioners as entitled to be heard in consultations, there could be no impediment to receiving them into the closer embrace of the family circle, and we are no longer left with the solution that straws show the course in which the wind blows, but may rest assured that birds of a feather will flock together."

THE ELIGIBILILY OF HOMŒOPATHIC GRADUATES TO MEMBERSHIP.—The minutes of a meeting of the Comitia Minora were read, showing that among the candidates for membership there were two gentlemen who were graduates of homœopathic colleges. To these candidates the following-named questions had been put:

1. Whether they belonged to a homœopathic medical society. They had answered, "No."

2. Whether they were willing to drop their sectarian name. They answered that they had never practiced under a sectarian name, and never expected to do so.

3. Whether they were connected in any way with any homœopathic medical journal. To this they had answered, "No."

4. Whether they were willing to be governed by the laws of the Medical Society of the County of New York. They had answered, "Yes." The Comitia therefore recommended them for membership.

Dr. A. Jacobi said that Dr. Ellsworth Eliot, who was unable to be present, had requested him to offer a resolution to the effect that a diploma from a homœopathic medical college did not entitle the holder to membership in the society.

The resolution was laid on the table.

The two candidates were then unanimously elected to membership,—*New York Medical Journal.*

